# Supplementary material for: Expression and Purification of BsaXI Restriction Endonuclease and Engineering New Specificity From BsaXI Specificity Subunit
Source: Front Microbiol. 2022 May 19;13:888435. doi: 10.3389/fmicb.2022.888435 (PMC9159934; doi:10.3389/fmicb.2022.888435)
Supplement: Supplementary file 1 [file Data_Sheet_1.PDF]

Supplementary Material

Supplement Figures S1-S4

Expression and Purification of BsaXI Restriction Endonuclease and Engineering New Specificity from BsaXI Specificity (S) Subunit

Sonal Gidwani^#, Daniel Heiter^, Shuang-yong Xu*

New England Biolabs, Inc. 240 County Road, Ipswich, MA 01938, USA

^ These authors contributed equally to this work

#Current address:

Calder Biosciences, Inc. 140 58th Street, Bldg A, Unit 8J, Brooklyn, NY 11220, USA

*Corresponding author
Email: xus@neb.com
Telephone: 1-978-380-7287

Suppl. Fig. S1. BsaXI TRD1-CR1 (top) and TRD2-CR2 (bottom) predicted secondary structure by the Phyre2. Green coil, α-helix; blue arrows, β-sheets. There are two predicted long α-helixes in TRD2-CR2, located at the N-terminus and C-terminus, respectively.
A and B, Amino acid sequence alignment of TRD1-CR1, TRD2-CR2 with Type I S subunit TRDs. **C**. BsaXI S structure model predicted by Phyre 2 and schematic diagram of BsaXI S and BsaXI RM subunits. **D**. Predicted 2xTRD1 (TRD1-CR1-TRD2-CR2) model by Phyre2 with schematic diagram of two RM subunits.

A.

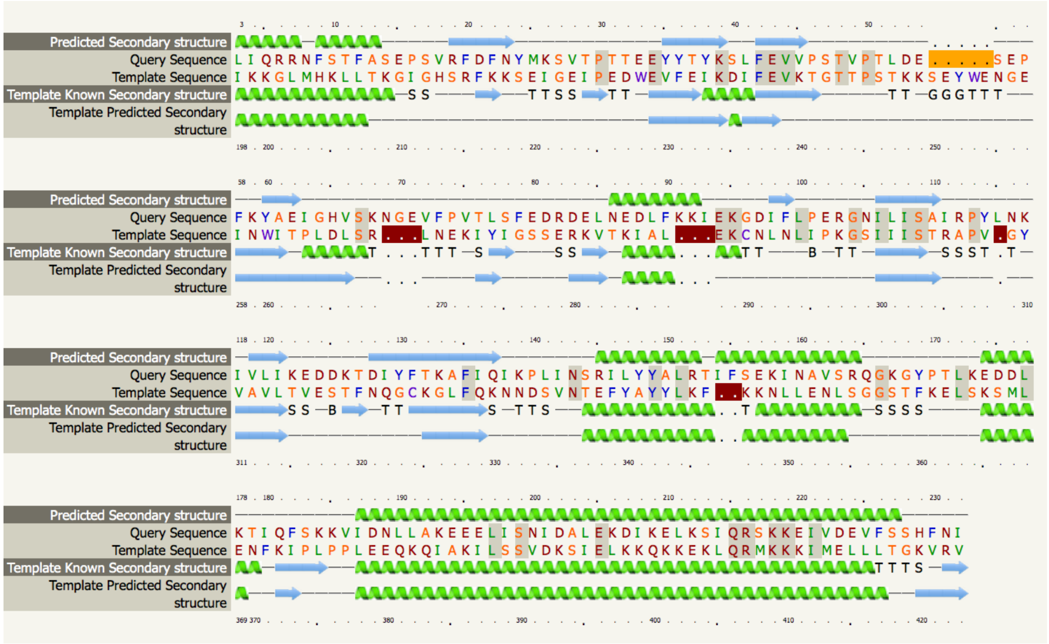

B.

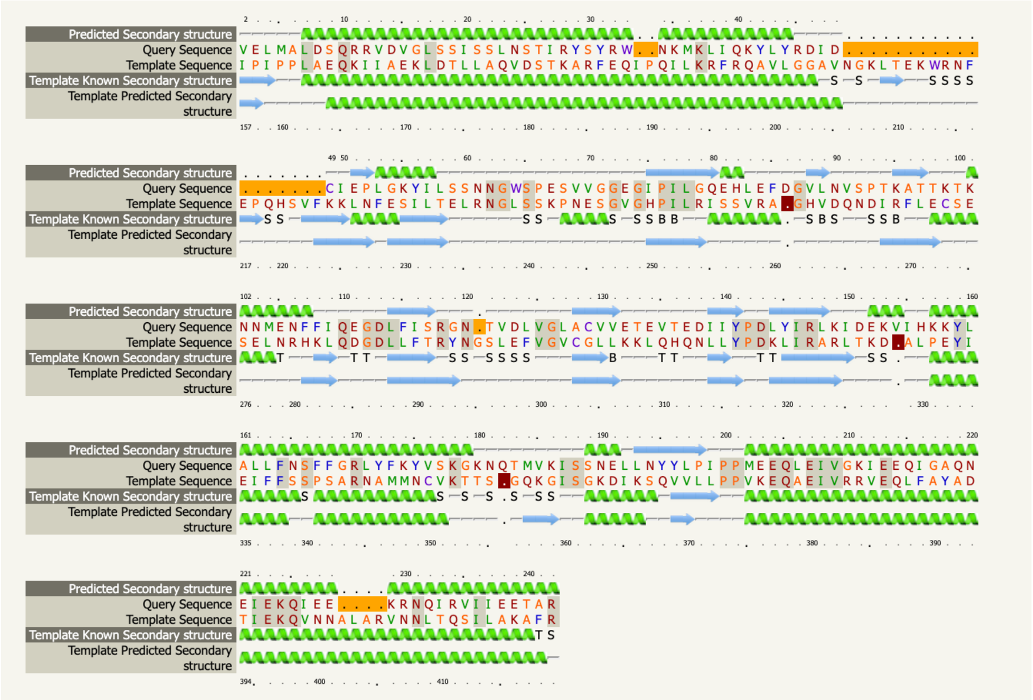

C.

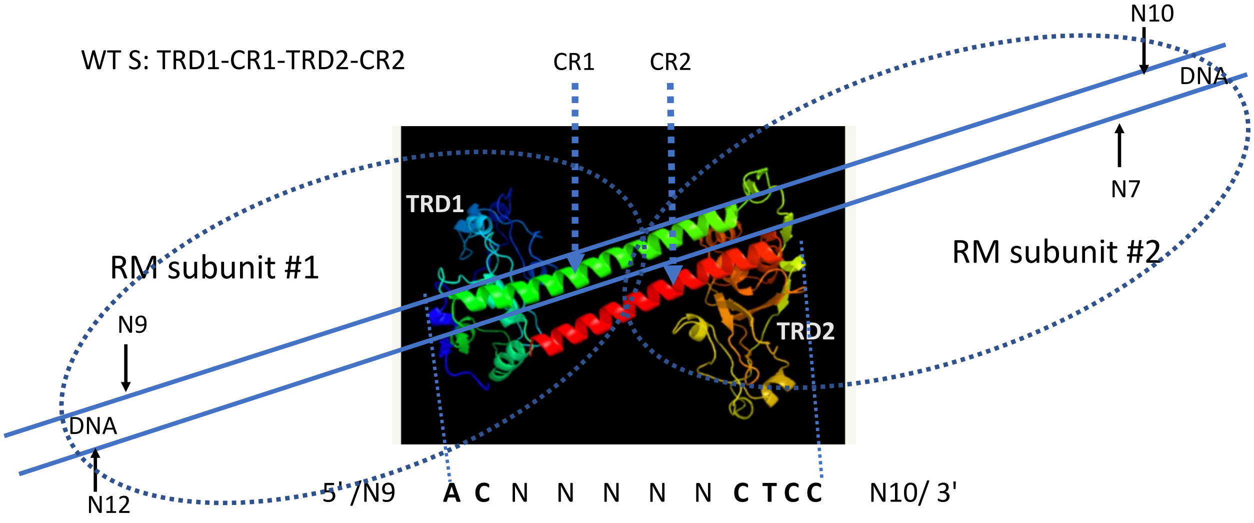
WT S: TRD1-CR1-TRD2-CR2
CR1
CR2
N10
DNA
N7
TRD1
RM subunit #1
RM subunit #2
TRD2
N9
DNA
N12
5' /N9 A C N N N N N C T C C N10/ 3'

D.

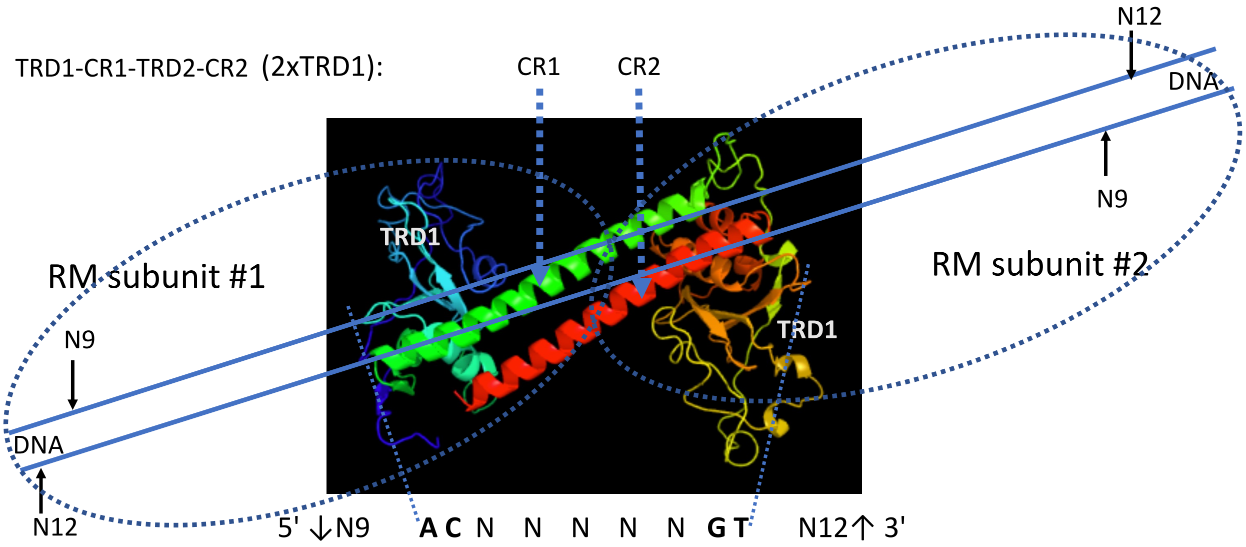
TRD1-CR1-TRD2-CR2 (2xTRD1):
CR1
CR2
N12
DNA
N9
TRD1
RM subunit #1
RM subunit #2
TRD1
N9
DNA
N12
5' ↓N9 A C N N N N N G T N12↑ 3'

Suppl. Fig. S2. SDS-PAGE analysis of partially purified BsaXI REase, RM, S, S (6xHis) subunits, and S subunit variants. M. protein molecular mass standard in kDa (NEB). Purified target protein is marked by an "*". BsaXI RM subunit was purified from chitin, DEAE, and Heparin columns; S (6xHis) protein was purified from a Ni agarose column; WT S and S subunit variants were purified from chitin columns by DTT cleavage of target-intein-CBD fusions.

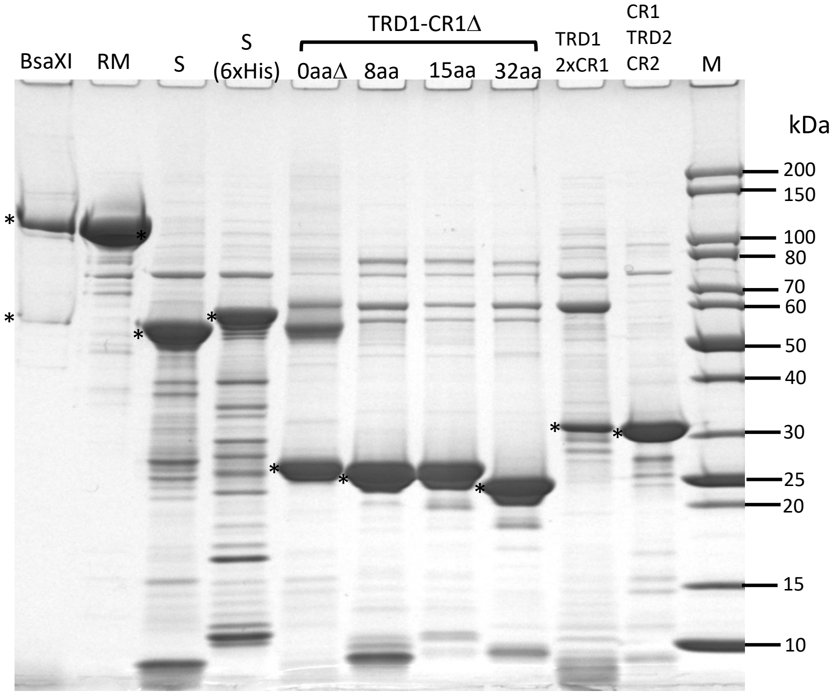

Suppl. Fig. S3. BsaXI restriction activity reconstituted by mixing purified RM (chitin/DEAE/Heparin columns) and S (6xHis) (Ni agarose column) subunits *in vitro* in restriction digestion of λ DNA. Fixed amount of RM subunit (1 μg, at ~93.5 nM, lanes 1-4; 2 μg, at ~187.0 nM, lanes 5-8) was mixed with varying amount of S (6xHis) (0.25, 0.5, 1, and 2 μg, at 90.9, 181.9, 363.6, and 727.2 nM). RM to S ratio approximately at 1/1, ½, ¼, and 1/8 in lanes 1-4; at 2/1, 1/1, 1/2 to 1/4 in lanes 5-8. Lanes 9-11, BsaXI RM subunit only; BsaXI positive control (4 U), uncut DNA. 2-log, DNA size ladder (0.1 to 10 kb, NEB).

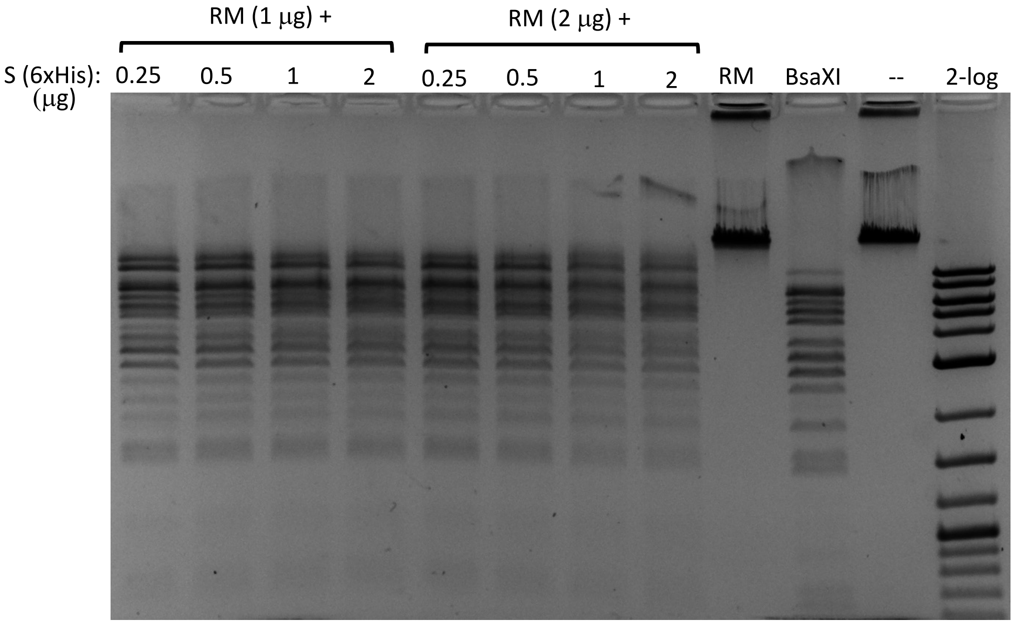

Suppl. Fig. S4. Cleavage site mapping of TRD variants in complex with RM subunits (TRD1-CR1, TRD1-2xCR1, TRD1-CR1-8aaΔ, TRD1-CR1-21aaΔ) by DNA run-off sequencing. A. Cleavage upstream (N12) and downstream (N9) of ACgcatcGT site in pBR322 by TRD1, TRD1-2xCR1, and two TRD1-CR1 deletion variants (sequencing panels 2 to 5) compared to undigested DNA (sequencing panel 1). **B**. Cleavage upstream of ACcaaacGT site by TRD1-CR1-21aaΔ. Cleavage took place mostly upstream and a low level of cleavage downstream as the result of asymmetric partial digestion. **C.** Possible cuts downstream of a star site **T**Ccttcg GTN9↑ and cleavage upstream of a cognate site ↑N12ACtcatgGT.

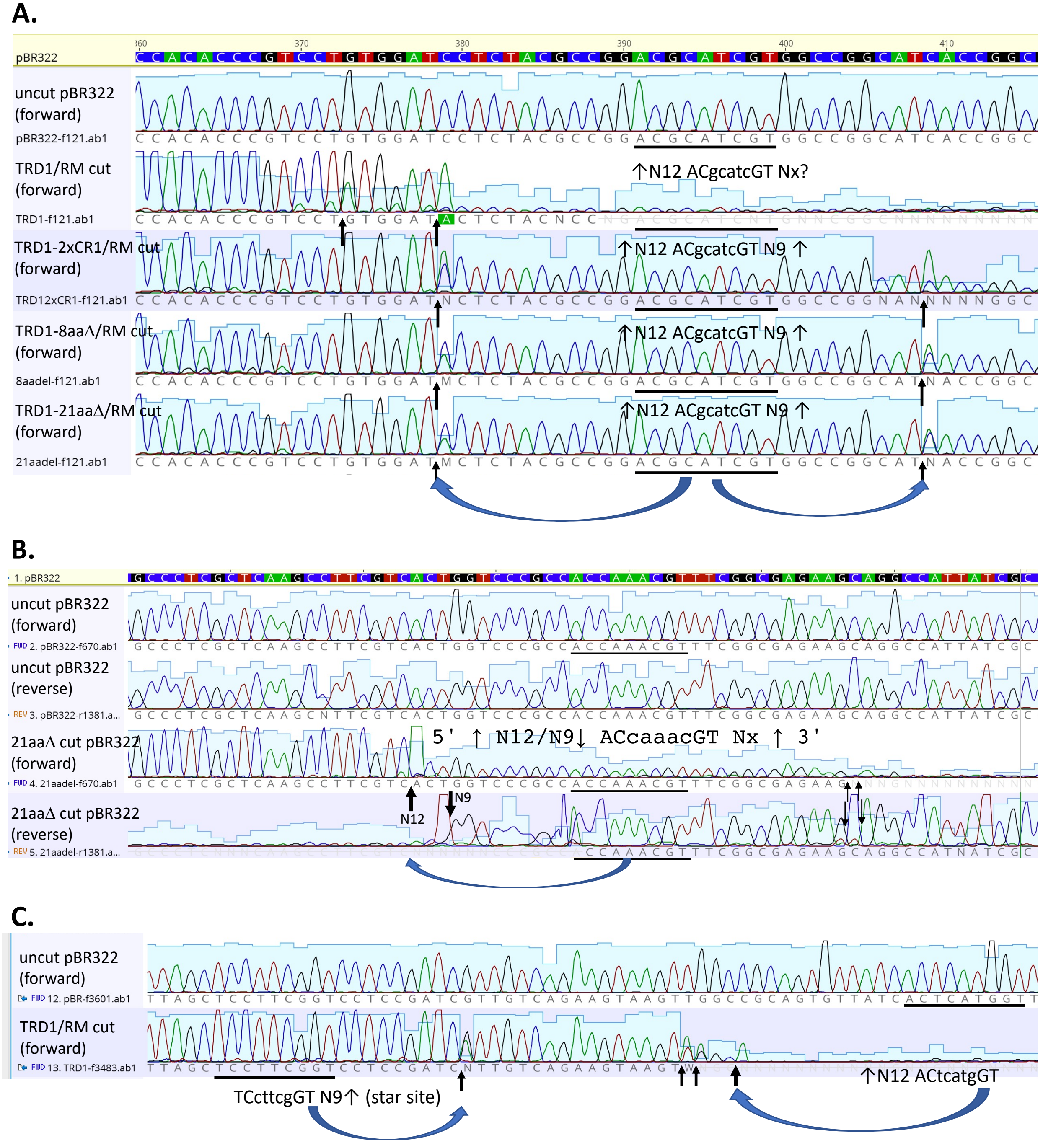

Suppl. Fig. S5. PROMALS3D multiple sequence alignment of 10 standalone (orphan) TRDs (BsaXI TRD2 homologs) from sequenced microbial genomes. The numbers (5-9) on top of aa sequences indicate the degree of conservation at the particular positions. Predicted α-helix = h; predicted β-sheet = e. The highly conserved aa residues are shown in bold letter. There are nine conserved Ile (I) and Leu (L) residues in the C-terminal CR2 region that are presumably important for interactions with CR1 and cognate RM subunits to form four helix bundles.

```
Conservation:                                                       7       77 65 559 97 6559
GciITRD2           1   -------MQKQSLFKSKIIDFSSNKDLRFSYKFHNKVAVFLKNFLVNQT-TKKIKNFTCEPIILGKGIKP    62
BthITRD2           1   ----------------------------MGIRFHNKAGVYIQSFLEKKT-NKRIKDFISEPIALGKSISP    41
HchITRD2           1   ---------------------------------------MNYLKSIT-NKKIKDFISEPIVLGKGILP    28
CbrTRD2            1   -MHAFEKEKKQRLQILNLSDFANNKDLRCGIRFHNPAYKFLHKYLTGLF-RTKIKDFISEPIVLGKGISP    68
CmoITRD2           1   ---------------------------------------MEQLEKIT-NKKIKHFISEPIVLGKSVSP    28
BbaGITRD2          1   ---------------------------------------MSVLKTQT-SKRIKDYTAEDITLGKGISP    28
CbaITRD2           1   ----------------------------MGLRFHNKAGKYLYSFLCSKT-KRNIKDFIDEPIVLGQSVSP    41
AacITRD2           1   ----------------------------MGVRFHNLAGEYLQSFLSKIS-NKKIKDFLAEPIVLGTSVSP    41
DspITRD2           1   ----------------------------MGVRFHNKAGAYIQSFLGSKT-NKRIKDFISEPIVLGKSVSP    41
BbaITRD2           1   ----------------------------MSYKFHNKAGKYVYDFLSHLT-TKRIKDFTEKPIVLGKSVSP    41
BsaXITRD2          1   MVELMALDSQRRVDVGLSSISSLNSTIRYSYRWN-KMKLIQKYLYRDIDCIEPLGKYILSS---NNGWSP    66
Consensus_aa:          .........................................b.hh.pbs..c.l.c@h.psIlLspthSP
Consensus_ss:                    hhhhhhh             eee       hh        eeee eeeeeee

Conservation:            77  7 557  75577  5 75 5 55 5   5   7 57  5 67755797777 779777575 5
GciITRD2          63   KEYDDEGEYFYFSMADIKQ-WKFNPEDCRRVNETFYLKNINKTVQLNDILLARSGEG-TIGKVALIDNDE   130
BthITRD2          42   SDYDEDGEYFYIAMSNIKS-WAFDPENCKKVSDSYAASNLNKTVKKGDILLARSGEG-TIGKVALIEDED   109
HchITRD2          29   RQYDDEGHYYYIAMSNIKK-WKFESEDCKKVSDSFYANNINKNIKLNDILIARSGEG-TIGKVALIEDDD    96
CbrTRD2           69   NDYDEEGKYYYISMADIKN-WYFNQEECKTVGDEFYRSNFNKSFAMNDIIMARSGEG-TIGKVAIIQDEE   136
CmoITRD2          29   SDYDDNGDYFYVSMANIKN-WKFESDDAKLISKEYSRQNENKTVAKGDILIARSGEG-TIGKVALIDDDE    96
BbaGITRD2         29   SDYDENGEQYYVSMADIKN-WRFEPEEAKKVFQSYFNANPNKRIAINDIIMARSGEG-TIGKVAIIDNDE    96
CbaITRD2          42   NDYDDEGEYFYIAMSNIKT-YAFEEEDCKKVSEEYALNNIKKIVKKGNILLARSGEG-TIGKVALIEDEE   109
AacITRD2          42   SDYDENGEYYYIAMSNIKT-WAFDDEDCKKVSDTYSQNNQNKTLQKNDILLARSGEG-TIGKVALIDVDE   109
DspITRD2          42   SDYDEDGEYFYIGMSNIKS-WAFDPEDCKKVSESYAASNLNKTVKKGDILLARSGEG-TIGKVAIIEDEE   109
BbaITRD2          42   KDYDKEGKYYYIAMSNIKT-WAFESEDCNRVNEIYWLENLHKTVQKNDILLARSGEG-TIGKVALIEEKN   109
BsaXITRD2         67   ESVVGGEGIPILGQEHLEFDGVLNVSPTKATTKTKN-NMENFFIQEGDLFISRGNTVDLVGLACVVETEV   135
Consensus_aa:          pshs....h.hltb.plc....hpspshK.hscpb..p.bNb.l.bsDlhltRtsps.hlGbhtll-s-.
Consensus_ss:          hh      eeeeeeeeee   eeee   ee  hhhhhhhhh       eeeeee       eeeee

Conservation:             556597559577    55 5 775775755775655 57997 77777977575  5755     59
GciITRD2         131   -WKGVFADFVMRIRLK--GYNPLLAYFYFQSDLFQYLVYTHKKGL-GNNTNIFPSQIQDFPILDLCVEKQ   196
BthITRD2         110   -INGIFADFTQRIRLN--DFDPLCAYYYMRSDFFQYLVYTHKKGL-GNNTNIFPSQIKEFPIPDWDEVKQ   175
HchITRD2          97   -VEGIFADFTMRVRLK--EYNPKFAYYYFRSELFQYLIYTNKKGL-GNNTNIFPGQIQELPILDFDQGQQ   162
CbrTRD2          137   -IEGLFADFTMRIRLR--NYNPLLAYYYFRSDFFQYYIYTHKKGL-GNNTNIFPSQVWEFPIPDFSEKKQ   202
CmoITRD2          97   -LQGVFADFTMRIRLK--NYNPLFAYYYFRTEYFQYLIEVNKKGL-GNNTNIFPSQIQELPMIDISLEKQ   162
BbaGITRD2         97   -NEGVYADFTMRIRLK--NYNPLFAYYYFRTDFFQHLIYTHKKGL-GNNTNIFPSQIQEFPIPELTIKQQ   162
CbaITRD2         110   -INAIFADFTQRIRLI--NYNVLLAYYYFRSDFFQYLVYMHKKGL-GNNTNIFPSQIQHFPIPDWEETKQ   175
AacITRD2         110   -INAIFSDFTQRIRLT--GFNPLCAYYYFRSDFFQYLIYIHKKGL-GNNTNIFPSQIQELPMPNWNKEKQ   175
DspITRD2         110   -INGIFADFTQRIRLT--GFDLLCAYYYMRSDFFQYLVYTHKKGL-GNNTNIFPSQIKEFPIPDWDDIKQ   175
BbaITRD2         110   -IKAIFADFTQRIRLT--NYNHRLAYYYFRSDLFQYLVYAHKKGL-GNNTNIFPSQIQEFPIPDWTMTKQ   175
BsaXITRD2        136   TEDIIYPDLYIRLKIDEKVIHKKYLALLFNSFFGRLYFKYVSKGKNQTMVKISSNELLNYYLPIPPMEEQ   205
Consensus_aa:          .bp.l@sDhhbRl+lp..shp.bhhhhhFpS.h.phhhbhhpKGb..s.hpI.ssplbph.l...sbpcQ
Consensus_ss:             eee  eeeeee    h  hhhhhhhh  hhhhhhhhhh            hhhhh        hhhh

Conservation:            77  9   6  9  5   9  7   9  795   5
GciITRD2         197   EMIIHEIKTQLEEQAEIQEKINMKKAEIIHIIEIESFDSETEIIN   241
BthITRD2         176   AEIVEKIRTQIDEQKGIDRQIEEKQQVINKIIEN-AIR-------   212
HchITRD2         163   TQIVNQIQAKINAQKVIDRQIREKCKEIEKIIKQ-AI--------   198
CbrTRD2          203   KSIVEKIKSELDKQKEIEREIDEKQKEISRIIEA-AIKG------   240
CmoITRD2         163   QKIVDEIKAKLDKQEEMKEKIESERNKIDEIIEK-AIK-------   199
BbaGITRD2        163   ENIVSEIKASIDAQREIEKQIQEKQQEISKIIID-AIKS------   200
CbaITRD2         176   NEIVLRIKTQIDAQNLVDLAIEAKQQEINEIIEN-AIKE------   213
AacITRD2         176   SEVVKKIKAELEKQQLIELQIEEKQKEISNLIEL-VIKQDPILDI   219
DspITRD2         176   AEIVAKIKMQLDEQKVIDRQIEEKQQAINKIIEE-AIKQGQ-RNA   218
BbaITRD2         176   NEIVKKIKTQIDVQRVVDSEIKKKQDEISQLIED-TIMK--EII-   216
BsaXITRD2        206   LEIVGKIEEQIGAQNEIEKQIEEKRNQIRVIIEE-TARS------   243
Consensus_aa:          .pIV.cIc.pls.Qp.l-ppIcpKpppIp.IIE..hh+.......
Consensus_ss:          hhhhhhhhhhhhhhhhhhhhhhhhhhhhhhhhhhh hh
```
